# EEG epilepsy seizure prediction: the post-processing stage as a chronology

**DOI:** 10.1038/s41598-023-50609-z

**Published:** 2024-01-03

**Authors:** Joana Batista, Mauro F. Pinto, Mariana Tavares, Fábio Lopes, Ana Oliveira, César Teixeira

**Affiliations:** 1 Center for Informatics and Systems of the University of Coimbra, Department of Informatics Engineering, University of Coimbra, Coimbra, Portugal; 2 Epilepsy Center, Department Neurosurgery, Medical Center–University of Freiburg , Faculty of Medicine, University of Freiburg, Freiburg, Germany

**Keywords:** Biomedical engineering, Epilepsy, Machine learning

## Abstract

Almost one-third of epileptic patients fail to achieve seizure control through anti-epileptic drug administration. In the scarcity of completely controlling a patient’s epilepsy, seizure prediction plays a significant role in clinical management and providing new therapeutic options such as warning or intervention devices. Seizure prediction algorithms aim to identify the preictal period that Electroencephalogram (EEG) signals can capture. However, this period is associated with substantial heterogeneity, varying among patients or even between seizures from the same patient. The present work proposes a patient-specific seizure prediction algorithm using post-processing techniques to explore the existence of a set of chronological events of brain activity that precedes epileptic seizures. The study was conducted with 37 patients with Temporal Lobe Epilepsy (TLE) from the EPILEPSIAE database. The designed methodology combines univariate linear features with a classifier based on Support Vector Machines (SVM) and two post-processing techniques to handle pre-seizure temporality in an easily explainable way, employing knowledge from network theory. In the Chronological Firing Power approach, we considered the preictal as a sequence of three brain activity events separated in time. In the Cumulative Firing Power approach, we assumed the preictal period as a sequence of three overlapping events. These methodologies were compared with a control approach based on the typical machine learning pipeline. We considered a Seizure Prediction horizon (SPH) of 5 mins and analyzed several values for the Seizure Occurrence Period (SOP) duration, between 10 and 55 mins. Our results showed that the Cumulative Firing Power approach may improve the seizure prediction performance. This new strategy performed above chance for 62% of patients, whereas the control approach only validated 49% of its models.

## Introduction

Epilepsy is one of the most common neurological diseases, affecting over 50 million people worldwide. This condition is expressed by atypical brain activity that results in seizures or unusual behavior, sensations, and sometimes loss of awareness. This abnormal activity leads to neurological, cognitive, psychological, and social consequences^1^. Despite the first-line treatment for epilepsy being Anti-Epileptic Drugs (AEDs), almost one-third of epileptic patients suffer from Drug-Resistant Epilepsy (DRE)^2^. In the scarcity of completely controlling a patient’s epilepsy, seizure prediction plays a significant role in clinical management and therapy. This field would attempt to improve the quality of life of patients who are susceptible to the sudden occurrence of seizures.

The seizure prediction field aims to develop an algorithm capable of anticipating an epileptic seizure by raising an alarm before the seizure onset. The goal is to design a system able to read online data and promptly notify the patient regarding a seizure that will arise on a well-defined occurrence period (Seizure Occurrence Period—SOP) with a predefined horizon (Seizure Prediction Horizon—SPH), allowing enough time to take action. An accurate system may provide new therapeutic options, such as warning devices that enable the patient to avoid dangerous situations or even intervention devices capable of controlling the seizure by delivering anti-convulsive drugs or triggering electric stimuli^3,4^.

This field has been moving forward by assuming the existence of a preictal period that Electroencephalogram (EEG) signals can capture^3^. The preictal is considered a transitional period that precedes the seizure. Beyond this period, the EEG signal can be divided into three more stages: ictal (the seizure), postictal (the period after the seizure), and interictal (the period between the postictal and preictal stages of two consecutive seizures)^5^.

Throughout the years, various seizure prediction studies have employed classical machine learning approaches. These classification algorithms follow general steps, including signal processing, feature extraction, classifier training, and post-processing^4,6,7^. Deep learning approaches have recently been applied since they can automatically learn more distinct and robust features and have an improved potential to deal with temporal and spatial dependencies^4,6–13^. Despite the ability of deep learning models to produce more accurate results, their lack of interpretability has raised some skepticism regarding their clinical applicability^14^.

After the classification, most studies^13,15–22^ conducted a regularization step to deal with the temporality of the data and algorithms’ decisions. The Kalman filter^23^ and the Firing Power^23^ are post-processing methods widely used in seizure prediction. However, this step is primarily used to reduce the number of false alarms and improve the classifier’s specificity. It does not offer any physiological insights into the process of seizure generation.

The preictal period also represents a significant challenge for seizure prediction. Usually, the preictal stage is viewed as a single event with a fixed duration. In some studies, its choice follows a grid search approach of different periods ranging from 10 to 75 mins^15,17,20,21^. However, this period is associated with substantial heterogeneity. Indeed, there is evidence that this stage may vary among patients and between seizures from the same patient^7,14^.

To deal with temporality and interpretability challenges, Pinto et al.^20,21^ published two works with interpretable patient-specific seizure prediction models based on evolutionary computation to search for discriminative features while trying to discover the best preictal time. In these studies, they constructed hyper-features and placed each chronologically in a timeline, allowing the analysis of a sequence of events with a given interval instead of the traditional investigation of feature alterations in that same interval. Therefore, these methodologies provide a deeper understanding of the seizure generation processes by giving interpretable insights based on the obtained results and analyzing a sequence of instants. However, this methodology is complex and not very intuitive regarding the physiological perspective of the preictal events.

To tackle the referred aspects, we embraced a different view assuming that the epileptic seizure is not the consequence of just a single event but rather a succession of events. With this study, we intend to highlight the nature of the brain as a biological system, so the preictal is seen as a process of generating seizures, i.e., as a cascade of events of the brain activity, chronologically ordered. This concept derives from the network theory, which supports that seizures arise from abnormal activity in a distributed network (an epileptic network) whose interactions extend over large brain regions involving the seizure-onset zone, its surroundings, and distant areas rather than the idea of a localized and well-defined epileptic focus^4,7^. Indeed, many studies have demonstrated that channels in more distant and even contralateral areas transmit the relevant information^24,25^.

Our approach attempts to improve the most common structure in seizure prediction techniques by explicitly incorporating the epileptic network concept. For this purpose, we developed patient-tailored algorithms for seizure prediction considering different post-processing methods based on the idea that a seizure is the consequence of a succession of critical brain events. We designed two methods: the Chronological Firing Power method, which presumes that a seizure is caused by three non-overlapping brain events, and the Cumulative Firing Power method, which views this process as three overlapping brain events. These methodologies were compared with the Control approach based on the most common seizure prediction pipeline, in which only one preictal event is assumed. The adopted preictal period ranges from 10 and 55 mins, in steps of 5 mins, with an intervention time of 5 mins. This way, based on existing brain knowledge, temporality can be applied quickly and understandably, overcoming the lack of interpretability of deep learning models.

## Methods

**Figure 1:**
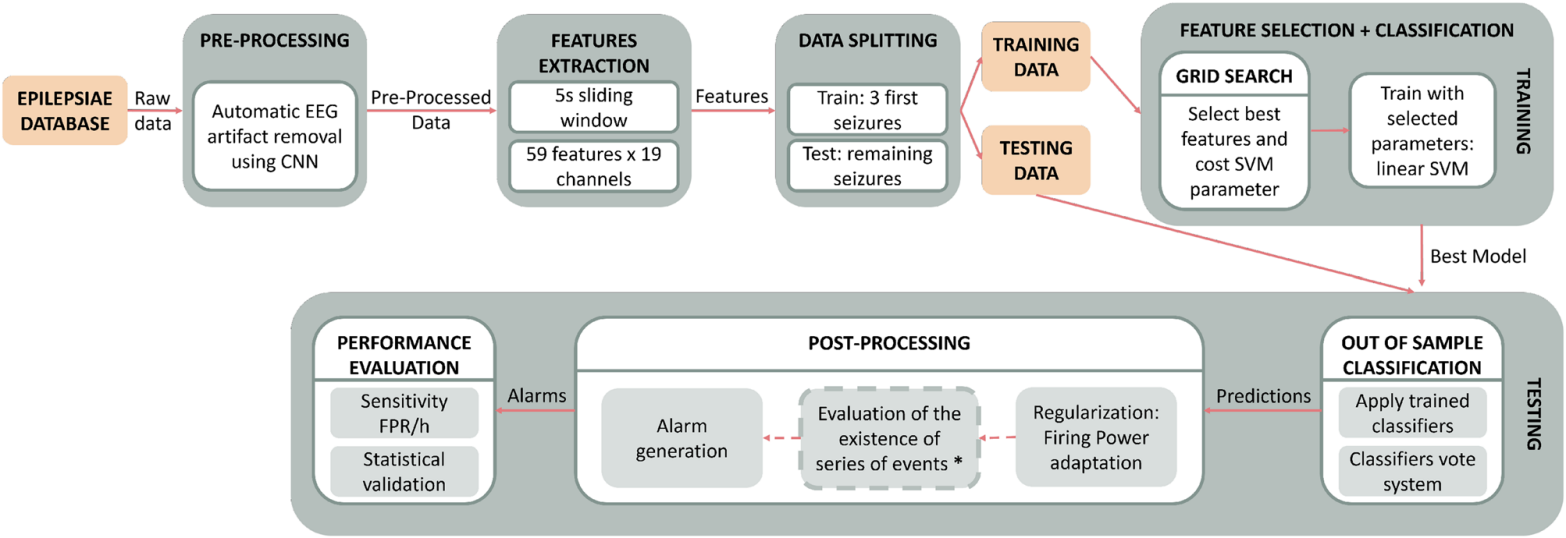
General outline of the proposed pipeline for each SOP and patient. * Only applied in Chronological and Cumulative Firing Power approaches.

We designed patient-specific algorithms for seizure prediction to analyze the influence of envisioning the chronology of brain activity as a post-processing stage. For this, two distinct methodologies were adopted, along with a control one based on the most common framework. As presented in Fig. 1, the procedure can be divided into data pre-processing, feature extraction, training, testing, and post-processing. The raw EEG data were pre-processed and segmented into non-overlapping 5-second windows to extract relevant features. Then, the data were divided into two groups: the training set used for parameters optimization and classifier training and the test set used to make predictions and evaluate the classifier. The post-processing phase was carried out by adapting the Firing Power method, and the chronological series of events were assessed. These algorithms were evaluated for several SOP values between 10 and 55 mins, with an SPH of 5 mins.

### Dataset

For the present study, we selected 37 Drug-Resistant Epilepsy (DRE) patients (17 females and 20 males, with a mean age of 41.0 ± 16.1 years) from the European Epilepsy Database (EPILEPSIAE)^26^. The usage of these data for research purposes has been approved by the Ethical Committee of the three hospitals involved in the EPILEPSIAE database development (Ethik-Kommission der Albert-Ludwigs-Univer sität, Freiburg; Comité consultatif sur le traitement de l’information en matière de recherche dans le domaine de la santé, Pitié- Salpêtrière University Hospital; and Comité de Ética do Centro Hospitalar e Universitário de Coimbra). All studies followed relevant guidelines and regulations. Informed written patient consent was also obtained.

The selected EEG data was collected by the University Medical Center of Freiburg, in Germany, from patients containing seizures localized in the temporal lobe, the most usual form of focal epilepsy^27^. The data consists of EEG scalp recordings acquired during pre-surgical monitoring. It covers 19 EEG electrodes placed according to the International 10–20 System. We selected the 37 patients based on the following criteria: (1) a minimum of four lead seizures, separated by at least 4.5 h to avoid analyzing clustered seizures; (2) EEG scalp recordings acquired with a sampling rate of 256 Hz. As a result, the total dataset includes approximately 5120 h of recording time containing 209 seizures.

### Pre-processing and feature extraction

The raw EEG data were pre-processed using an EEG artifact removal model based on deep convolutional neural networks (DCNN). This model was proposed by Lopes et al.^28^ to automatically and quickly remove artifacts from EEG signals, mimicking the manual pre-processing performed by experts. They used long-term EEG recordings from patients in the EPILEPSIAE database, including data used in the present work.

After the pre-processing phase, the EEG signals were segmented into 5-second windows without overlap to extract relevant features from the data. We used a sliding window analysis for each channel to compute univariate linear features from time and frequency domains since they present a relatively lower computational power^7,13,15,19–22,29^. Regarding the frequency domain features, we extracted the absolute and relative spectral power of the following bands: delta (0.5–4 Hz), theta (4–8 Hz), alpha (8–13 Hz), beta (13–30 Hz), and four gamma sub-bands—gamma band 1 (30–47 Hz), gamma band 2 (53–75 Hz), gamma band 3 (75–97 Hz), and gamma band 4 (103–128 Hz); the ratio between these spectral band powers, spectral edge frequency and power at 50%, the alpha peak frequency, the total power, and the mean frequency. For the time domain, we computed the four statistical moments (mean, variance, skewness, kurtosis), the Hjörth parameters (activity, mobility, complexity), and the decorrelation time. Concerning the time-frequency features, we extracted the wavelet coefficients’ energy by performing wavelet decomposition with the Daubechies 4 mother wavelet. As a result, 59 linear univariate features were computed.

For each patient, we divided the dataset into two distinct groups: the training set, constituted by the first three seizures and used for parameters optimization and classifier training, and the test set, composed of the remaining seizures and used to evaluate the classifier. Therefore, 111 seizures ($\approx 3660$ h) were used in the training phase, and 98 ($\approx 1460$ h) were applied in the testing phase.

### Training

The data samples were labeled into two classes: preictal (class 1) and interictal (class 0). Regarding the preictal category, several SOP values were analyzed at intervals of 5 minutes between 10 and 55 minutes. A 55-minute maximum duration was defined since patients often prefer preictal periods shorter than 1 h^30^. The SPH value was set to 5 min since it is considered a suitable time interval for patients to minimize seizure consequences.

As seizures are relatively rare events, there is a significant imbalance between interictal and preictal samples. Therefore, we implemented a systematic random undersampling to balance the data^13,20–22^. For each seizure, we divided the interictal set into *n* groups, corresponding to the total length of the preictal class, and randomly selected one sample from each group. This way, we cover the overall interictal period.

After the class balancing stage, we normalized the range of independent features extracted from the raw data. Then, we selected the *k* most discriminative features employing the ANOVA (Analysis of Variance) f-test^31^. Regarding the most suitable number of features to be chosen (*k*), we applied a grid-search procedure to tune this parameter.

We used the SVM (Support Vector Machines) model with a linear kernel for the classification stage. It only involves the optimization of one parameter, the cost parameter (*C*), which was tuned using a grid-search procedure. Due to the stochasticity intrinsic to the random undersampling performed during the class balancing, we implemented an ensemble learning approach. In this procedure, 31 SVM classifiers were trained with different data samples.

We adopted a grid-search strategy to find the optimal parameters to train the classifier. It included the search for the most suitable number of features (*k*), the appropriate value for the SVM hyperparameter (*C*), and the most suitable preictal period (*SOP*). For parameter *k*, we considered four different values (10, 20, 30, and 40 features), and for *C*, eleven distinct values (2^-10^, 2^-8^, 2^-6^, 2^-4^, 2^-2^, 2^0^, 2^2^, 2^4^, 2^6^, 2^8^, 2^10^). Thus, 44 combinations (*k*, *C*) were evaluated for each SOP value. We implemented the Leave-One-Out Cross-Validation strategy to find the optimal parameters. Therefore, considering the training set, we employed a 3-fold cross-validation using two seizures to train and one for validation. We used a performance metric that examines the trade-off between sample sensitivity ($SS_{sample}$) and sample specificity ($SP_{sample}$) to evaluate each model: $\sqrt{SS_{sample} \times SP_{sample}}$. After assessing all combinations (*k*, *C*, SOP), we selected the highest-performing one. Finally, we trained an ensemble of 31 classifiers using the best parameters and the entire training set.

A training procedure pseudo-code is included in the Supplementary Material in the Training Methodology section.

### Testing

After training the model, an out-of-sample classification was applied to the testing set. The procedure used for the testing data was the same as the training set, excluding the class balancing. The testing set was standardized, using the z-score parameters of the training set, and the most relevant features identified in training were selected. Finally, we employed the trained SVM classifier to determine the output.

This procedure was executed for each of the 31 trained classifiers, resulting in 31 predictions per sample. We employed a voting system strategy: we assigned the most voted class as the final output for each sample.

### Post-processing

After classification, we performed a regularization step to reduce the number of false alarms and noise. In this stage, we introduced two new strategies to deal with the pre-seizure temporal dynamics. We implemented an adaptation of the Firing Power method proposed by Teixeira et al.^23^. This method consists of a sliding window technique that computes the ratio of preictal samples. The Firing Power is defined by:

1 $$\begin{aligned} fp[n] = \frac{\sum _{k=n-\tau }^{n} O[k]}{\tau } \end{aligned}$$

Where *fp*[*n*] is the firing power regularization output that ranges between 0 and 1, $\tau$ is the number of samples of the moving window, with size equal to the preictal period, and *O*[*k*] is the binary classifier output at instant *k*.

An alarm is triggered when the *fp*[*n*] value exceeds a predefined threshold and is separated by at least one refractory period from the last generated one. This period reduces the patient’s stress and anxiety, minimizing the consecutive alarms during the same seizure. We considered a firing power threshold of 0.5 and a refractory period corresponding to the preictal’s total duration (SOP + SPH).

As mentioned, the present study intends to embrace the brain as a biological system. Thus, we developed two approaches, the Chronological Firing Power approach and the Cumulative Firing Power approach, which assumed the generation of seizures as a sequence of events in brain activity. Each method is based on a succession of three events since we aimed to select a reasonably substantial number of events for the proposal while keeping the approaches simple and intuitive. Depending on the trained classifier, different post-processing techniques were also applied. The Control approach was used for comparison.

#### Control

**Figure 2:**
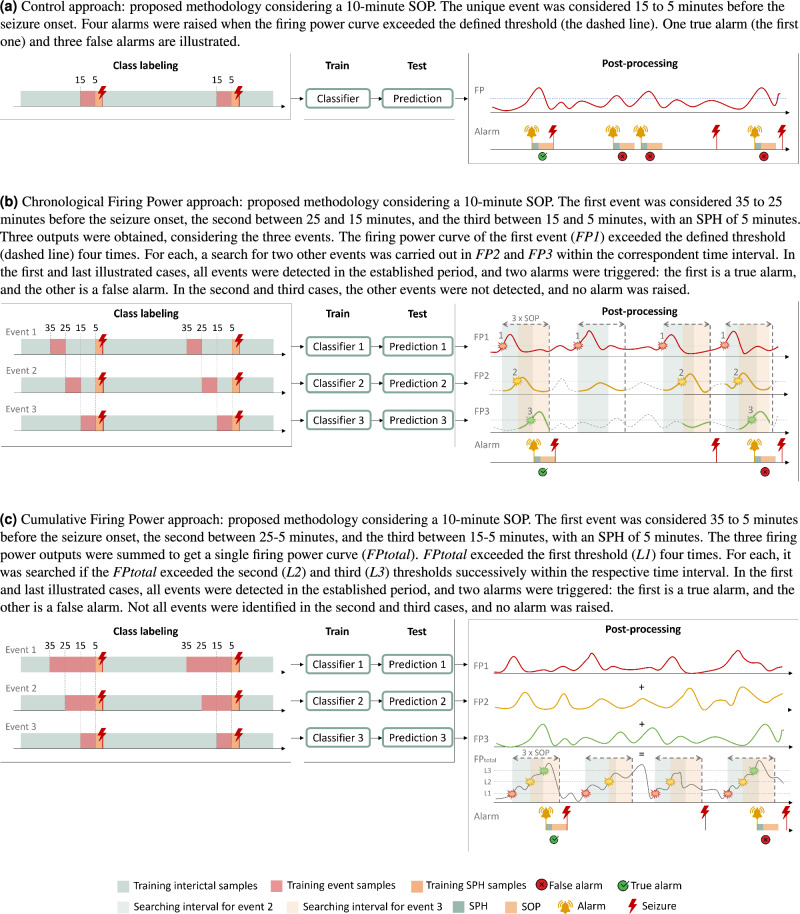
General overview of this study’s framework applied in the three approaches.

The Control approach, represented in Fig. 2a, simulates the most used strategy in the epileptic seizure prediction field. It considers the preictal as a unique event with equal duration to the adopted SOP. Therefore, following the described pipeline, only one model output is obtained. After that, the post-processing procedure is applied, and the performance is evaluated.

#### Chronological Firing Power

In the Chronological Firing Power approach, we assumed that a seizure is the consequence of a succession of physiological events of brain activity chronologically ordered and separated in time. We considered three non-overlapping sequential events, each with a duration equal to the adopted SOP value, as represented in Fig. 2b. Considering the different class labels for the three events, we applied the described pipeline to each one, resulting in three regularization curves. During the post-processing, we assumed a given event occurred when the respective firing power curve exceeded the established threshold of 0.5. Therefore, an alarm was triggered when the three events occurred chronologically within the stipulated time interval.

#### Cumulative Firing Power

In the Cumulative Firing Power approach, we assumed that a seizure is the consequence of a series of physiological events of brain activity chronologically ordered and overlapping in time. We considered three events of different duration, as represented in Fig. 2c. The third event, the closest to the seizure onset, was shorter, with an equal period to the SOP value. The second and first events were two and three times longer than the third. Regarding the different class labels for the three events, we applied the described pipeline to each one, resulting in three regularization curves. The three firing power curves were summed to form a single firing power curve used to evaluate the existence of a chronological sequence of events. To trigger an alarm, we considered three different thresholds (0.5, 1, 1.5) that the firing power curve must exceed sequentially, from the smallest to the largest, in the stipulated time. Therefore, after the threshold of 0.5 is exceeded, it is necessary that the points of 1.0 and 1.5 also be overpassed in the given time to raise an alarm.

### Performance

To evaluate the performance of the seizure prediction models, we used the standard metrics—seizure sensitivity (SS) and false positive rate per hour (FPR/h)—and a statistical validation strategy^3,7^. A seizure is correctly predicted when its onset occurs after an alarm and within the established SOP. Otherwise, the alarm is assumed to be a false alarm.

Seizure sensitivity measures the fraction of the correctly predicted seizures, as described by equation (2).

2 $$\begin{aligned} SS = \frac{Predicted \ seizures}{All \ seizures} \end{aligned}$$

FPR/h measures the occurrence of false alarms during an hour and is defined as the proportion between the number of false alarms and the period during which they can be raised (the interictal period excluding the refractory period), as expressed by equation (3).

3 $$\begin{aligned} FPR/h = \frac{False \ alarms}{Interictal \ duration - False \ alarms \times Refractory \ duration} \end{aligned}$$

Along with the performance assessment, we also implemented a statistical validation strategy using the seizure-times surrogates method. This procedure was conducted to confirm if the developed algorithm performed above the chance level, i.e., if the model effectively identified the preictal stage and was not merely a matter of chance^32,33^. The seizure-times surrogates method was carried out seizure by seizure, randomly shifting the original onset time to another location within the interictal period. The developed algorithm performs better than chance if its seizure sensitivity is higher than the surrogates one and statistically significant, considering a significance level of 0.05 under the following null hypothesis: the seizure sensitivity of the proposed methodology is not superior to the seizure sensitivity of the surrogate predictor.

We also performed pairwise hypothesis tests to compare the different developed approaches. We employed Tukey’s Honest Significant (HSD) test^34^ to determine whether the means of SS and FPR/h values statistically differ between methods. This statistical test is used to adjust for multiple comparisons. We tested the hypothesis that the means of each pair of groups are equal against the general alternative that they are not equal, considering a significance level of $\alpha =$ 0.05.

## Results

We started by analyzing the overall results and comparing the different approaches. Following, we examined the individual patient results for each approach.

### Overall performance of seizure prediction models

**Figure 3:**
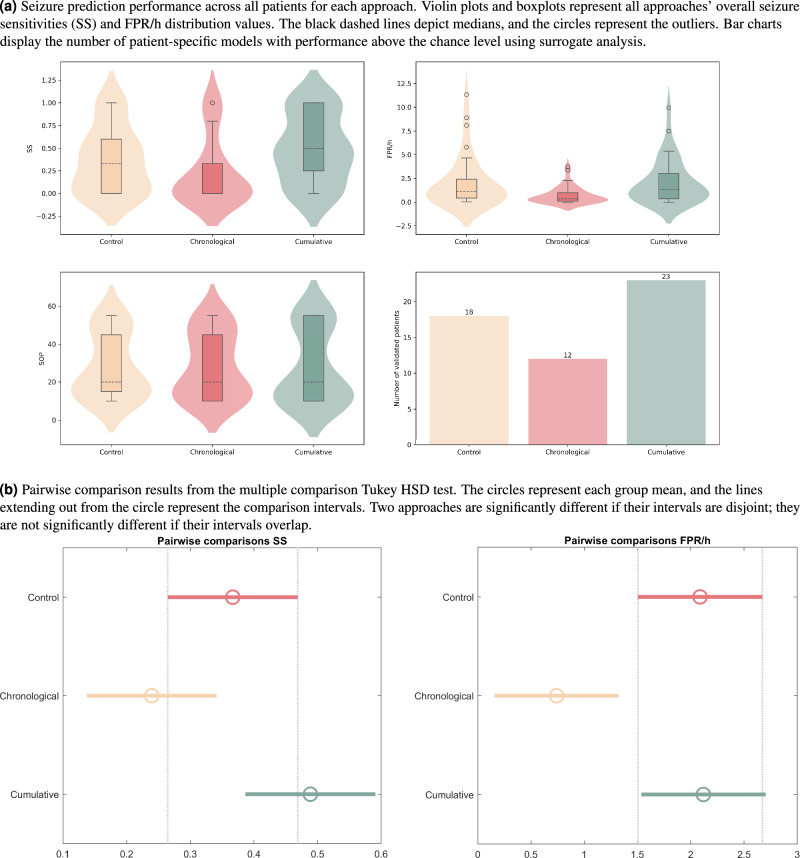
Performance comparison between approaches.

Table 1 presents the average seizure prediction results for the three developed approaches. Figure 3a displays violin plots with boxplot overlays illustrating overall seizure sensitivity (SS) and FPR/h values for all approaches. There’s also a bar chart indicating the number of models performing above the chance level. Figure 3b summarizes the results obtained for pairwise statistical comparison.

As demonstrated in Table 1, the Cumulative Firing Power approach presents a higher average seizure sensitivity than the other methods. Observing the violin plots for SS values (Fig. 3a), we can confirm that the Chronological Firing Power approach shows a null median, suggesting that at least 50% of its models cannot predict seizures. Indeed, an analysis of the data distribution reveals that the seizure sensitivity values for this approach are predominantly concentrated around 0. The maximum SS value for this method is 0.8, showing five outliers with a value of 1. These outliers emphasize that seizure sensitivity for this approach predominantly clusters in lower values, implying that the Chronological Firing Power approach may not be effective in predicting a high number of seizures. It suggests that the methodology successfully captures subtle patterns mainly in these five subjects. Concerning the Control approach, we can observe that 75% of the patients have a seizure sensitivity inferior to 0.6, and at least 25% of the models present a null seizure sensitivity. A closer look at the distribution of SS values suggests that 0 is the most frequent value. However, some points also cluster around 0.5 and 1. In contrast, the Cumulative Firing Power approach shows a more pronounced concentration in higher values of seizure sensitivity, with at least 25% of the models having a seizure sensitivity value of 1. Furthermore, it is evident from the distribution that the SS values are more concentrated around the median (0.5). Analyzing the results obtained in the pairwise comparisons for seizure sensitivity (Fig. 3b), we can conclude that the Cumulative Firing Power approach only shows SS values significantly higher than the Chronological Firing Power approach (p-value = 0.01).

As shown in Table 1, the Chronological Firing Power approach presents the best average FPR/h value. Furthermore, upon examining the violin plots for FPR/h values (Fig. 3a), it becomes evident that this approach exhibits a higher concentration in lower values than the other two methods. The distribution curve reveals that the most frequent values cluster around the median in all approaches. Additionally, when considering a sequence of events preceding the seizure, a decrease in the number of outliers becomes evident. It suggests that the newly designed approaches yield fewer outliers, characterized by lower values. However, some outliers persist, which indicates that the atypical elevated number of false alarms may be attributed to artifacts or noisy data. As depicted in Fig. 3b, the FPR/h values in the Chronological Firing Power approach are significantly lower than in the other methods (p-value = 0.02). However, the average values of FPR/h for Control and Cumulative Firing Power approaches are similar, not presenting statistically significant discrepancies (p-value = 1.00).

Regarding the statistical validation (Table 1 and Fig. 3a), the Cumulative Firing Power approach is the one that produced a higher number of models ($\approx 62\%$) performing above the chance level. The Chronological Firing Power approach presented the lowest percentage of statistical validation ($\approx 32\%$ of the models), and the Control approach could also not achieve statistical validation for at least 50% of the patients.

Concerning the chosen SOP values (Table 1 and Fig. 3a), they differ considerably between patients and approaches. However, the average value across the 37 patients is similar between methods: the average SOP value is inferior to 30 mins for all methodologies. Analyzing the violin plots it is noticeable that all approaches exhibit the same median (20 mins) and a binomial distribution. The first distribution peak occurs between 10 and 20 mins and the second one around 50/55 mins. The last peak is more evident in Control and Cumulative Firing Power approaches.

**Table 1:** Average seizure prediction performance for all approaches, considering the 37 patients.

Approach	SOP	SS	FPR/h	Statistical Validation
Control	28.11 ± 16.62	0.37 ± 0.36	2.09 ± 2.66	18 48.65%
Chronological	28.24 ± 16.89	0.24 ± 0.37	0.74 ± 0.89	12 32.43%
Cumulative	27.70 ± 19.16	0.49 ± 0.37	2.12 ± 2.28	23 62.16%

### Individual performance of seizure prediction models

**Figure 4:**
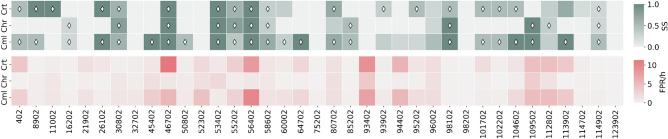
Seizure prediction sensitivity (SS), FPR/h, and results of statistical validation of all post-processing approaches for each patient: Crt (Control), Chr (Chronological Firing Power), and Cml (Cumulative Firing Power). The top subfigure represents the seizure sensitivity values obtained for each patient-specific model, and the bottom subfigure depicts the obtained FPR/h values. The color scales are defined in the color bars on the right side of each subfigure. The diamond shape indicates the models that performed above the chance level.

Figure 4 illustrates seizure sensitivity and FPR/h for each patient using a color gradient. The figure also highlights models that performed above the chance level.

Analyzing the obtained results for each patient (Fig. 4), it is possible to conclude that whereas 27 patients ($\approx 73\%$) were statistically validated by at least one approach, ten patients ($\approx 27\%$) were not statistically validated by any of the three approaches.

Considering the validated patients, the Control approach led to better results for six of them ($\approx 16\%$), out of which three were only statistically validated by this method. The Chronological Firing Power approach outperformed the other techniques for seven patients ($\approx 19\%$), from which one performed above the chance level only with this approach. For nine patients ($\approx 24\%$), the Cumulative Firing Power approach performed better than the others. Five of those models were statistically validated only by this method. For four patients ($\approx 11\%$), determining the best approach can be challenging since they present two well-performed approaches. Whereas one approach offers the best results regarding seizure sensitivity, the other minimizes the false alarms more effectively. In such cases, the choice of the optimal approach should be guided by the requirements of the final prediction system. For one patient (patient 98102), the Chronological and Cumulative Firing Power approaches obtained the same results.

When comparing the new methodologies with the Control one, the rate of false alarms decreased in most patients ($\approx 78\%$), the seizure sensitivity increased in fifteen patients ($\approx 41\%$), and the developed models became statistically valid for nine patients ($\approx 24\%$). On the other hand, for eight patients ($\approx 22\%$), all approaches simultaneously performed above the chance level.

Detailed results are provided in the supplementary material (Table S2, S3, S4, and S5).

## Discussion

### Performance of seizure prediction models

The Cumulative Firing Power approach is a less restrictive method for raising alarms. It only requires that the sum of the three firing power curves exceeds a threshold of 1.5 within the stipulated time interval to raise an alarm, instead of relying on the chronological detection of the three overlapping events. Therefore, it presents the highest average seizure sensitivity, and, consequently, the most elevated percentage of statistically validated models. However, on average, this method continues to predict less than half of the patients’ seizures ($\approx 49\%$), which may not be a suitable value for real-life applications. Additionally, with the analysis in the Results section, it is possible to infer that when we consider the temporality of the seizure generation, despite this approach presenting the highest average SS values, the seizure sensitivity values do not show statistically significant improvements compared to the Control approach. Furthermore, the elevated FPR/h average value obtained with this approach may also lead to questioning the applicability of the developed system in real life since the high rate of false alarms per hour may bring consequences to the patient’s health, such as unnecessary interventions, increased anxiety levels, and distrust of the warning systems^3^. However, other applications, such as neurostimulation closed loop systems, may have a higher tolerance toward false alarm interventions^35^. This pipeline offers an advantage over the other methodologies regarding the models’ statistical validation since more than half of its models performed above the chance level.

The Chronological Firing Power approach is the most restrictive and conservative since the three events must be detected sequentially in a given time interval to trigger an alarm, presenting a decreased seizure sensitivity. Consequently, it also offers fewer statistically validated models since patient-specific models with null seizure sensitivity could not be validated using surrogate analysis. As a result of being more conservative, this pipeline presents fewer false alarms. Regarding the FPR/h, this method is more advantageous for some patients and more suitable for some clinical applications, such as warning devices and rescue medication usage. However, the average FPR/h value is still superior to 0.15, the maximum false positive rate value considered acceptable in pre-surgical monitoring data. Suitable performance must be defined respecting the patient, chosen intervention system, and clinical considerations^3^.

Regarding the optimal SOP, the average value is inferior to 30 mins for all methodologies, comprising a suitable time interval since most patients prefer relatively short seizure prediction windows^30^. Furthermore, interventions that last the whole seizure occurrence period may increase the risk of undesirable consequences to the patient’s health if too long SOP values are considered. In the case of a warning system, long seizure occurrence periods may also increase the patient’s stress and anxiety^3^. The standard deviation of the average SOP values is relatively high, proving that the chosen SOP value varies extensively among patients. These results reflect the heterogeneity of the seizure generation process.

### Firing power plots analysis

We also analyzed the firing power plots over time for each patient since this evaluation can provide more insights into the models’ performance and the classifiers’ dynamics. By inspecting the firing power functions, it is possible to identify some patterns in the models’ behavior^22^.

**Figure 5:**
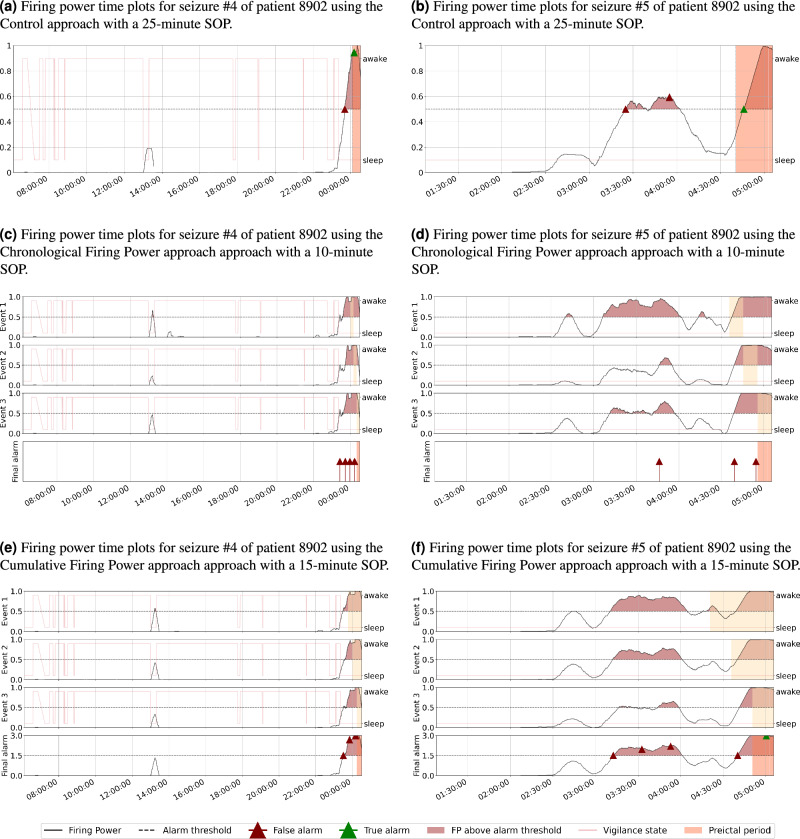
Firing power time plots of patient 8902 for the three approaches.

We found that the developed methodologies present a similar dynamic in most models. However, there are some patients for whom the regularization curves show slight differences among the approaches. The Chronological Firing Power approach is the most distinctive. It was already expected since this is the most distant approach from the Control: the first two events occur farther from the seizure onset. Accordingly, the first event is the most distinctive, while the third is similar to the control approach. On the other hand, the variability of the SOP duration among techniques also contributes to classifiers’ dynamic differences: in general, higher SOP values result in a flatter regularization curve, while lower SOPs result in a sharper firing power function. The referred aspects can be observed in the examples present in Fig. 5.

It was also possible to identify cases where no true alarm was raised despite the respective firing power curve being above the defined threshold. As depicted in Fig. 5c–e, the firing power curves are above the threshold near the seizure onset and during the respective event period. However, despite the classifiers presenting an expected behavior, the seizures were not predicted because the alarms were raised a few moments before the period defined as preictal. It led us to speculate that if we considered higher SOP values, the seizure could have been predicted. These examples demonstrate how controversial the selection of the most suitable SOP value can be and its critical role in classifier performance.

### Other studies comparison

The results obtained with the proposed approaches can be compared with other seizure prediction studies^13,16,20–22^ that employed data from the EPILEPSIAE database and implemented statistical validation. Most of these authors used a higher prediction horizon (10 mins). However, we considered that 5 mins is a suitable time interval for patients to prepare for the coming seizure. For instance, considering an alarm system in which, ideally, the patient would have enough time to take some rescue medication before the seizure, such as intranasal Diazepam, approved by the FDA (Food and Drug Administration) in 2020, it would take 5 mins to work^36^. In some other studies^17–19^, various parameters, such as the preictal duration, are selected according to the best testing performance. It may lead to a bias in the presented results and an impeding of real-life applications once the model parameters are chosen based on the test results that are unknown a priori. Therefore, they cannot be directly compared to our methodology since our model parameters were selected based on defined training metrics.

Alvarado Rojas et al.^16^ used 53 patients from the EPILEPSIAE database. They developed a threshold classifier to investigate the interactions between the phase of low-frequency rhythms (slow waves and theta) and the amplitude of different sub-bands of gamma rhythms. Our Cumulative Firing Power approach outperformed their model in seizure sensitivity (0.47), while our Chronological Firing Power method reached better results in terms of FPR/h (0.94). Regarding the statistical evaluation, their model only performed above the chance level in 13% of the patients, a validation percentage inferior to our proposed approaches. However, this comparison is limited since they used a different statistical validation method: the random predictor.

Pinto et al.^20,21^ published two studies with seizure prediction models based on evolutionary computation. In this study, we used data from some patients included in both Pinto et al., works. Both papers employed an SPH of 10 mins and a set of SOPs between 20 and 50 mins. As in the present work, they analyzed a sequence of events instead of only one instant. However, whereas they examined different hyper-features ordered chronologically in a timeline, here we considered three independent events, dealing with the temporality only in the post-processing stage. In the first study^20^, they used data from 19 patients and obtained performance above the chance level in 32% of the models. They achieved an average seizure sensitivity of $0.40 \pm 0.23$ and an average FPR/h of $0.71 \pm 0.40$. In the second study^21^, they used data from 93 patients and obtained performance above the chance level in 32% of the cases. They reached an average SS of $0.16 \pm 0.11$ and an average FPR/h of $0.21 \pm 0.08$. Regarding the average SS value, at least one of our approaches presents better results than both Pinto et al. studies. However, their models show an average FPR/h value better than our approaches. Finally, our models performed above chance in an equal or superior percentage of the patients.

Lopes et al.^13^ explored two critical aspects of seizure prediction model development: the effect of performing a robust pre-processing to remove noisy artifacts from EEG signals; and the impact of periodically retraining the models to manage possible concept drifts. The importance of these two concepts was investigated for a deep neural network using EEG time series as input and for a shallow neural network utilizing handcrafted EEG features as input, resulting in eight distinct approaches. They employed data from 41 patients, including those in the present study. Also, we used the pre-processing methodology as outlined in their research. Their proposed approaches obtained average SS values between 0.13 and 0.37 and average FPR/h values between 0.24 and 0.93. Comparing the performances, it is observable that at least one of our approaches shows better results regarding the SS average values. However, regarding the FPR/h metric, Lopes et al. presented more promising results in most approaches. Concerning the statistical evaluation, their models performed above the chance level in 20% to 54% of the patients, depending on the method used. This value is inferior to the percentage of models statistically validated by our Cumulative Firing Power approach.

In another study, Pinto et al.^22^ tried to evaluate and comprehend what may be the crucial model explanations for EEG seizure prediction. To this end, they developed three machine learning methodologies with different transparency levels to explore their explainability potential: a logistic regression, an ensemble of 15 support vector machines, and an ensemble of three convolutional neural networks. They used 40 patients from the EPILEPSIAE database, including all the patients employed in the present study. The pre-processing procedure was also the same one used in our research. Depending on the pipeline used, the average values varied between 0.04 and 0.13 for seizure sensitivity, 0.18 and 0.87 for FPR/h, and the percentage of validated models ranged between 7.5 and 17.5. Therefore, our approaches outperformed their methodologies in SS and statistically validated models. However, regarding FPR/h, generally, their models achieved better performances.

These findings show that it is possible to address the temporality of the seizure generation process easily and understandably, performing above the chance level for a higher ratio of patients ($\approx 62\%$) compared to other more complex methods. However, our Chronological Firing Power approach is clearly outperformed regarding FPR/h values.

### Study limitations

The present study has some limitations that should be addressed. The first limitation concerns using EEG data collected from patients during pre-surgical monitoring, which does not reflect actual seizure activity. Due to time constraints, patients suffer from medication tapering and sleep deprivation, resulting in an artificially high seizure frequency. Furthermore, the activities of patients under pre-surgical monitoring may be quite different from those of regular daily routine since they are mostly seated or lying down. This altered state may promote notable variations in the data distribution, affecting the efficiency of the trained prediction models. Therefore, taking care before generalizing the results to real-life applications is crucial^3,14,26^.

Another constraint is the reduced number of evaluated seizures per patient, which ranges from 1 to 5. Eight patients present only one testing seizure, 12 present two seizures, and 17 have more than two evaluated seizures. For patients with only one testing seizure, the seizure sensitivity is limited to 0 (seizure not predicted) or 1 (seizure correctly predicted). These discrepancies in possible seizure sensitivity values are responsible for the large standard deviations, which might influence the statistical comparisons. Furthermore, these differences make the comparison between patients difficult. A more detailed analysis regarding the influence of the number of testing seizures on model performance is presented in the Discussion section within the Supplementary Material.

Regarding these limitations, long-term EEG recordings, comprising several months or years, acquired in an everyday routine, represent a step forward in the clinical viability of the designed methodologies.

Finally, our models are designed to raise alarms whenever they identify any preictal changes in the data, not measuring seizure susceptibility over time. In other words, our models present an “all-or-nothing” response: even if the classifier accurately predicts the seizure, if the alarm is triggered just a second before the preictal, it would be evaluated as a false alarm (Fig. 5c–e). In that way, seizure forecasting may improve this limitation since it aims to determine periods of a high probability of seizure occurrence instead of just alarms, presenting a continuous response^37^.

## Conclusion

With this study, we intended to explore the seizure generation process as a chronological sequence of events in brain activity in a post-processing stage. Therefore, we proposed patient-specific seizure prediction algorithms to improve the most common seizure prediction methodologies explicitly and interpretably. To this end, we developed two approaches considering the preictal as a succession of three events and distinct post-processing methods. In the Chronological Firing Power approach, we considered three non-overlapping events that must be detected chronologically within the stipulated time interval to trigger an alarm. In the Cumulative Firing Power method, we assumed three overlapping events whose the combined firing power curves must surpass three predetermined thresholds to trigger an alarm. These methodologies were compared to the Control approach, in which seizures are assumed to be a consequence of a unique event fixed in time.

The Chronological Firing Power approach presents statistically substantial advantages over the Control approach, considering the false positive rate. However, although it is a logical and intuitive approach, this methodology is too conservative in triggering alarms. The Cumulative Firing Power approach presents a considerable advantage over the Control one regarding the statistical evaluation: it validates five more patients than the Control method.

Therefore, the Cumulative Firing Power approach concerns an improvement regarding the typical seizure prediction pipeline. However, it is essential to emphasize that our methods’ performance is still far from ideal for a real-life application.

In future work, the developed methodologies should be replicated in ultra-long-term data collected from daily life conditions, such as those performed by Cook et al.^38^.

### Supplementary Information

Supplementary Information.

## Data Availability

The data used in this study are not publicly available. However, the data can be made available from the corresponding author upon reasonable request and with permission from the EPILEPSIAE Consortium. The code used in this study is available for public use at https://github.com/JoanaFBatista/EEG-Epilepsy-Seizure-Prediction-The-Post-processing-Stage-as-a-Chronology.
