# Supplementary material for: EEG epilepsy seizure prediction: the post-processing stage as a chronology
Source: Sci Rep. 2024 Jan 3;14:407. doi: 10.1038/s41598-023-50609-z (PMC10764904; doi:10.1038/s41598-023-50609-z)
Supplement: Supplementary file 1 — Supplementary Information. [file 41598_2023_50609_MOESM1_ESM.pdf]

Supplementary material for:

EEG Epilepsy Seizure Prediction: The Post-processing Stage as a Chronology

Joana Batista, Mauro F. Pinto, Mariana Tavares, Fábio Lopes, Ana Oliveira, César Teixeira

1 Patient and seizure metadata

Table S1 contains information regarding the group of patients with temporal lobe drug-resistant epilepsy from the EPILEPSIAE database[1] analyzed in this study. It presents information about each patient (gender, age, and number of seizures) and their seizures (seizure classification, seizure activity pattern, state of vigilance at seizure onset, and recording time).

Table S1: Dataset description regarding the 37 studied patients.

Patient ID	Age	Sex	Number of seizures (train/test)	Seizure classification	Seizure activity pattern	Vigilance at seizure onset	Recording duration (h)
402	55	f	3	FOIA, FBTC, FOIA	t, t, t	A, A, A	103.81
			2	FBTC, FOIA	t, t	A, A	29.66
8902	67	f	3	UC, FOIA, FOIA	a, b, a	A, A, A	133.91
			2	FOIA, FOIA	m, a	A, A	22.5
11002	41	m	3	UC, FOIA, FOIA	?, s, a	A, R, A	97.16
			1	FOIA	t	A	11.7
16202	46	f	3	UC, FBTC, UC	r, ?, r	A, A, A	201.32
			4	FOIA, FOIA, FOIA, FOIA	r, r, ?, r	A, A, A, A	34.45
21902	47	m	3	UC, FOIA, FOIA	t, t, t	A, A, A	67.08
			1	FOIA	b	R	9.76
26102	65	m	3	FOIA, FOIA, FOIA	m, t, t	A, A, A	60.65
			1	FOIA	t	A	22.58
30802	28	m	3	FOA, FOA, FOA	t ,t ,t	R, A, 2	87.57
			5	FOA, FOA, FOA, FOA, FOA	t, t, t, t, t	A, A, R, 2, 2	61.71
32702	62	f	3	FOIA, FOIA, FOIA	t ,t ,t	A, A, A	117.38
			2	FOIA, FOIA	r, a	A, A	20.49
45402	41	f	3	FOIA, FOIA, FOA	t, t, t	A, A, A	71.98
			1	FOIA	t	A	22.31
46702	15	f	3	FOA, FOIA, FOIA	a, a, t	A, 2, A	47.46
			2	FBTC, FOIA	b, t	2, A	12.6
50802	43	m	3	FOIA, UC, UC	t, t, t	A, 2, 2	165.93
			2	FOIA, FBTC	t, t	2, A	35.6
52302	61	f	3	UC, FOA, UC	?, ?, d	A, A, 1	76.45
			1	UC	t	A	6.85
53402	39	m	3	FOA, FOA, FOA	?, ?, ?	A, 2, A	70.31
			1	FOIA	t	A	13.73
55202	17	f	3	FOIA, FOIA, FOA	t, d, t	A, A, A	47.05
			5	UC, UC, FOA, UC, FOIA	t, t, t, r, r	A, A, A, A, A	65.37
56402	47	m	3	UC, UC, UC	t, ?, ?	A, A, A	184.22
			1	FBTC	a	A	20.25
58602	32	m	3	FOIA, FOIA, FOIA	r, t, t	A, R, A	96.94
			3	FOIA, FOIA, FOIA	r, r, t	A, A, 2	23.34
60002	55	m	3	FOIA, FOIA, FOIA	d, c, t	1, A, A	208.11
			3	UC, FOIA, FOIA	t, d, d	R, R, 1	152.4
64702	51	m	3	FOA, FBTC, FBTC	?, m, t	A, A, A	75.91
			2	FBTC, FBTC	t, t	A, 2	31.59
75202	13	m	3	FOA, FOA, UC	t, t, t	2, 2, A	100.94
			4	FOA, FOA, FOA, FOA	t, t, ?, t	A, A, A, A	52.63
80702	22	f	3	FOIA, FOIA, UC	b, b, ?	A, A, A	49.4
			3	FOIA, FBTC, FOIA	c, c, c	A, A, A	29.55
85202	54	f	3	FOIA, FOIA, UC	m, c, m	2, A, A	53.49
			2	UC, UC	m, m	A, A	20.42

Table S1: Dataset description regarding the 37 studied patients.

Patient ID	Age	Sex	Number of seizures (train/test)	Seizure classification	Seizure activity pattern	Vigilance at seizure onset	Recording duration (h)
93402	67	m	3	FBTC, FOIA, FOIA	t, t, t	2, 2, 2	98.0
			2	UC, UC	t, t	2, 2	54.07
93902	50	m	3	FOA, FOIA, FBTC	t, t, d	A, A, 2	370.83
			3	FOIA, FOIA, UC	d, d, d	A, 2, A	20.29
94402	37	f	3	FOA, UC, FOIA	?, d, b	A, A, A	120.23
			4	UC, FOA, UC, FOA	t, ?, b, ?	2, A ,2, A	30.37
95202	50	f	3	FBTC, FOIA, FOIA	b, b, b	2, 2, 2	57.6
			4	FOIA, UC, FOIA, UC	m, b, b, t	2, 2, 2, 2	89.53
96002	58	m	3	FOIA, FOIA, FOIA	t, t, t	A, A, A	48.4
			4	FOIA, UC ,FOIA, FOIA	d ,a ,t ,a	A, A, A, A	82.2
98102	36	m	3	FOA, UC, UC	?, ?, ?	A, A, A	108.61
			2	UC, FBTC	?, ?	A, A	45.68
98202	39	m	3	FOIA, FOIA, FOIA	t, a, t	A, A, A	111.33
			5	FBTC, FOIA, FOIA, FOIA, UC	t, t, t, t, t	A, A, A, A, A	49.88
101702	52	m	3	FOIA, FOIA, FOIA	t, t, t	A, A, A	28.41
			2	FOIA, FOIA	r, r	2, A	23.83
102202	17	m	3	FOA, UC, FOIA	b, ?, t	2, A, 2	57.45
			4	UC, FOA, FOIA, UC	?, t, t, t	A, A, 2, A	51.41
104602	17	f	3	FOIA, FBTC, FBTC	t, a, t	A, 2, 2	87.87
			2	FBTC, UC	t, d	2, 2	15.25
109502	50	m	3	FOIA, FOIA, UC	t, t, t	A, A, A	76.8
			1	UC	t	A	41.94
112802	52	m	3	UC, FOIA, UC	t, t, t	A, A, A	71.58
			3	FOIA, FOIA, UC	t, t, t	A, A, A	111.5
113902	29	f	3	UC, FOIA, FOIA	t, d, t	A, A, 2	61.98
			3	FOIA, UC, FOIA	t, t, t	A, 2, A	22.73
114702	22	f	3	FOIA, FOIA, UC	t, t, t	A, A, A	68.39
			5	FOIA, FOIA, FOIA, FOIA, FOIA	t, d, t, d, t	A, A, A, A, A	34.04
114902	16	f	3	FOA, FOIA, FOIA	s, b, s	A, A, A	26.55
			4	FBTC, UC, FOIA, FOIA	t, r, a, t	2, A, A, A	50.66
123902	25	f	3	FBTC, FBTC, FOIA	t, t, t	2, 2, R	152.11
			2	FOIA, FOA	t, t	A, A	30.15

Gender: female (f), male (m); Seizure classification: unclassified (UC), Focal Onset Aware (FOA), Focal Onset Impaired (FOIA), Focal to Bilateral Tonic-Clonic (FBTC); Seizure activity pattern: unclear (?), rhythmic sharp waves (s), alpha waves (a), rhythmic delta waves (d),rhythmic theta waves (t), rhythmic beta waves (b), repetitive spiking (r), cessation of interictal activity (c), amplitude depression (m); Vigilance state: awake (A), REM sleep stage (R), Non-REM sleep stage I (1), Non-REM sleep stage II (2).

2 Training methodology

Algorithm 1 Pseudocode illustrating the training procedure for each approach and patient.

```
Input: training_data ← first 3 seizures
Output: SVM_models ← ensemble of 31 SVM models
[best_SOP, best_k, best_C] ← gridSearch(training_data)
target ← classLabeling(training_data, best_SOP)
for classifier_i in [0, 1, ..., 30] do                                  ▷ 31 classifiers
    training_data ← classBalancing(training_data)
    training_data ← dataStandardization(training_data)
    training_data ← featureSelection(training_data, best_k)
    SVM_model ← SVM.train(training_data, target, best_C)
end for
```

Algorithm 2 Pseudocode illustrating the grid-search procedure for parameter selection using a leave-one-out cross-validation strategy.

```
Input: training_data ← first 3 seizures
Output: [best_k, best_SOP, best_C] ← optimal parameters combination considering the highest metric performance
SOP ∈ {10, 15, 20, ..., 55}                                             ▷ SOP values
k ∈ {10, 20, 30, 40}                                                    ▷ number of selected features
C ∈ {2^-10, 2^-8, ..., 2^10}                                            ▷ SVM Cost values
for each combination (SOP, k, C) do
    target ← classLabeling(training_data, SOP)
    for fold_i in [0, 1, 2] do                                          ▷ 3-fold cross-validation
        for classifier_i in [0, 1, ..., 30] do                          ▷ 31 classifiers
            [training_data, validation_data, training_target, validation_target] ← splittingData(training_data)
            [training_data, training_target] ← classBalancing(training_data, training_target)
            [training_data, validation_data] ← dataStandardization(training_data, validation_data)
            [training_data, validation_data] ← featureSelection(training_data, validation_data, k)
            SVM_model ← SVM.train(training_data, training_target, C)
            prediction ← SVM_model.predict(validation_data)
            performance ← prediction.evaluate(validation_target)
        end for
        final_performance ← mean(performances)      ▷ mean performance for each combination (SOP,
                                                      k, C) considering the 31 classifiers
    end for
end for
[best_SOP, best_k, best_C] ← choosingBestParameters(final_performance)   ▷ optimal parameters combination con-
                                                                           sidering the highest final_performance
```

3 Features description

Here are present some details regarding the extracted features. Linear features are mathematical measures that capture linear dynamics from the signal, using its phase/frequency and amplitude information. The EEG signal is assumed as quasi-stationary within each time window when this type of feature is extracted.

Statistical Moments

Statistical moments are widely used in seizure prediction studies to characterize the signal's amplitude distribution. The four moments are mean, variance, skewness, which measures the degree of asymmetries of the amplitude distribution, and kurtosis, which measures the relative flatness or peakedness of the amplitude distribution. The preictal period has been associated with considerable changes in these measures compared to the interictal period. In particular, a decrease in variance and an increase in kurtosis were observed in the preictal phase[2–6].

Hjörth Parameters

The Hjörth parameters consider standard deviations to quantify the dynamical signal properties. These are three time-domain measures of brain activity: activity, a measure of mean power, mobility, a measure of root-mean-squared frequency, and complexity, a measure of root-mean-square frequency spread. With the proximity to the seizure onset, an increase in mobility and complexity measures is observed[2,3,5–7].

Decorrelation Time

The decorrelation time is described as the first zero crossing of the autocorrelation function. It is an estimator of the data periodicity and the strength of linear correlations. The lower its values, the less the signal is correlated. Before seizures, a decrease in the decorrelation time has been reported[3,5].

Relative Spectral Power

The spectral power quantifies the signal power associated with specific frequency ranges. It is possible to compute the power spectral density (PSD) by applying the Fast Fourier Transform (FFT) to the EEG time series and then average the squared coefficients of the frequency range of interest.

In turn, the relative spectral power is characterized as the power of a given frequency band divided by the total power of the EEG signal. A normalized spectral power provides a more robust measure since there is more power in low frequencies than at high frequencies. Some authors have reported a transference of power from the lower to higher frequencies before the seizure onset[3,5–7].

Spectral Edge Frequency and Power

SEF (Spectral Edge Frequency) is commonly described as the minimum frequency below which a given percentage of the total power of the signal is contained. The SEP (Spectral Edge Power) is the value of the power existing below the defined threshold.

Regarding the EEG signal, most of the spectral power is comprised in the 0.5–40Hz band, and SEF 50 and SEP 50 are commonly used. SEF 50 is the frequency below which 50% of the total power of the signal up to 40 Hz is located, and SEP 50 is the corresponding power below the spectral edge frequency. Thus, SEF may be capable of capturing the dynamics mentioned above during the preictal[3,5].

Wavelet Coefficients Energy

The DWT (Discrete Wavelet Transform) is a time-frequency domain transform that can be an alternative to the FFT. It is capable of revealing the spectral and temporal properties of the signal. The wavelet transform decomposes the signal in different resolution levels according to specific frequency components. The first decomposition levels are associated with higher frequencies, while the last levels represent the lower frequencies. After the signal decomposition, it is possible to compute discriminant measures from distinct frequency bands by applying the wavelet coefficients. The quantification of the energy in different frequency ranges is an example of a feature that can be obtained using the wavelet transform[3,5].

4 Results

Table S2 contains the seizure prediction results obtained for each patient and approach, including the optimal SOP value, seizure sensitivities (SS), false prediction rate per hour (FPR/h), and the models performing above the chance level. Table S3 presents the statistical test results for each patient and approach, using the seizure-times surrogates method. Tables S5 and S4 include the multiple comparison results using the Tukey's Honest Significant (HSD) Test.

Table S2: Seizure prediction results obtained for each patient and approach.

Patient	Tested Seizures	Control				Chronological				Cumulative			
		SOP	SS	FPR/h	Above Chance	SOP	SS	FPR/h	Above Chance	SOP	SS	FPR/h	Above Chance
402	2	10	0.50	3.92	●	50	0.00	0.10		10	0.50	3.03	●
8902	2	25	1.00	0.16	●	10	0.00	0.36		15	0.50	0.38	●
11002	1	25	1.00	0.46	●	10	0.00	1.00		10	0.00	1.15	
16202	4	15	0.00	0.21		45	0.25	0.11	●	55	0.25	0.21	●
21902	1	20	0.00	1.61		15	0.00	0.36		20	0.00	1.35	
26102	1	55	1.00	1.02	●	50	0.00	0.16		55	1.00	0.39	●
30802	5	55	0.60	0.43	●	20	0.80	0.99	●	20	0.60	1.16	●
32702	2	20	0.00	0.45		10	0.00	0.49		10	0.00	1.01	
45402	1	50	0.00	1.15		10	0.00	1.65		15	1.00	3.41	●
46702	2	10	1.00	11.34	●	20	1.00	1.00	●	10	1.00	5.38	●
50802	2	20	0.00	0.47		10	0.00	0.59		10	0.50	0.79	●
52302	1	55	0.00	2.20		20	0.00	1.64		20	0.00	4.56	
53402	1	55	1.00	0.86	●	20	1.00	2.29	●	50	1.00	0.84	●
55202	5	10	0.60	3.23	●	55	0.80	0.53	●	10	0.40	2.91	●
56402	1	15	1.00	8.10	●	10	1.00	0.96	●	10	1.00	9.96	●
58602	3	15	0.33	1.21	●	35	0.33	0.93	●	55	0.67	1.42	●
60002	3	20	0.33	0.81		30	0.00	0.11		10	0.33	1.55	●
64702	2	10	0.00	2.42		40	0.00	0.20		10	1.00	2.37	●
75202	4	35	0.00	0.11		30	0.00	0.09		35	0.00	0.22	
80702	3	50	0.67	1.49	●	55	0.33	1.65		55	0.67	1.68	●
85202	2	20	0.00	0.17		55	0.50	0.13	●	55	0.50	0.20	●
93402	2	15	0.00	8.91		55	0.00	3.73		25	0.00	7.52	
93902	3	45	0.33	0.46	●	20	0.00	0.06		25	0.33	0.76	
94402	4	10	0.00	5.81		20	0.00	0.54		10	0.25	5.39	
95202	4	15	0.50	1.16	●	10	0.00	0.24		10	0.25	1.74	
96002	4	50	0.25	1.72		40	0.25	1.64		55	0.50	1.91	
98102	2	40	0.50	0.22	●	55	1.00	0.13	●	55	1.00	0.13	●
98202	5	15	0.00	0.05		10	0.00	0.00		10	0.00	0.00	
101702	2	10	0.50	1.99	●	35	0.00	0.34		55	0.50	0.49	●
102202	4	55	0.50	0.28	●	45	0.00	0.02		55	0.50	0.32	●
104602	2	25	0.50	1.28	●	50	0.00	0.30		10	1.00	2.18	●
109502	1	10	0.00	4.58		40	1.00	3.38	●	55	1.00	4.90	●
112802	3	10	0.33	4.68		15	0.33	1.33	●	10	0.33	4.90	
113902	3	50	0.67	3.75		20	0.00	0.00		45	1.00	3.69	●
114702	5	40	0.20	0.47		10	0.00	0.21		35	0.00	0.24	
114902	4	35	0.25	0.07	●	10	0.25	0.11	●	15	0.50	0.27	●
123902	2	25	0.00	0.04		10	0.00	0.04		15	0.00	0.04	
**Total**	98	-	-	-	18 48.65%	-	-	-	12 32.43%	-	-	-	23 62.16%
**Average ± s.d.**	-	28.11 ± 16.62	0.37 ± 0.36	2.09 ± 2.66	-	28.24 ± 16.89	0.24 ± 0.37	0.74 ± 0.89	-	27.70 ± 19.16	0.49 ± 0.37	2.12 ± 2.28	-

Table S3: Results obtained with the statistical validation using the seizure-times surrogates method.

Patient	Control					Chronological					Cumulative				
	SS	SS Surrogate	t statistic	p value	Above Chance	SS	SS Surrogate	t statistic	p value	Above Chance	SS	SS Surrogate	t statistic	p value	Above Chance
402	0.50	0.08 ± 0.19	-12.04	0.00	●	0.00	0.02 ± 0.09	1.00	0.84		0.50	0.018 ± 0.24	-7.08	0.00	●
8902	1.00	0.13 ± 0.22	-21.11	0.00	●	0.00	0.08 ± 0.23	1.98	0.97		0.50	0.13 ± 0.22	-8.93	0.00	●
11002	1.00	0.23 ± 0.42	-9.76	0.00	●	0.00	0.07 ± 0.25	1.44	0.92		0.00	0.30 ± 0.53	3.07	1.00	
16202	0.00	0.05 ± 0.12	2.26	0.98		0.25	0.03 ± 0.08	-13.73	0.00	●	0.25	0.10 ± 0.12	-6.60	0.00	●
21902	0.00	0.33 ± 0.47	3.81	1.00		0.00	0.17 ± 0.37	2.41	0.99		0.00	0.20 ± 0.48	2.26	0.98	
26102	1.00	0.37 ± 0.48	-7.08	0.00	●	0.00	0.17 ± 0.37	2.41	0.99		1.00	0.20 ± 0.40	-10.77	0.00	●
30802	0.60	0.44 ± 0.11	-7.95	0.00	●	0.80	0.39 ± 0.14	-15.30	0.00	●	0.60	0.39 ± 0.15	-7.90	0.00	●
32702	0.00	0.07 ± 0.17	2.11	0.98		0.00	0.7 ± 0.2	2.69	0.99		0.00	0.13 ± 0.22	3.25	1.00	
45402	0.00	0.50 ± 0.56	4.78	1.00		0.00	0.13 ± 0.34	2.11	0.98		1.00	0.57 ± 0.56	-4.18	0.00	●
46702	1.00	0.48 ± 0.35	-7.88	0.00	●	1.00	0.08 ± 0.19	-26.49	0.00	●	1.00	0.35 ± 0.37	-9.50	0.00	●
50802	0.00	0.05 ± 0.15	1.80	0.96		0.00	0.08 ± 0.19	2.41	0.99		0.50	0.10 ± 0.20	-10.77	0.00	●
52302	0.00	0.30 ± 0.46	3.53	1.00		0.00	0.10 ± 0.30	1.80	0.96		0.00	0.17 ± 0.37	2.41	0.99	
53402	1.00	0.20 ± 0.4	-10.77	0.00	●	1.00	0.43 ± 0.50	-6.16	0.00	●	1.00	0.37 ± 0.48	-7.08	0.00	●
55202	0.60	0.32 ± 0.17	-8.97	0.00	●	0.80	0.38 ± 0.17	-13.04	0.00	●	0.40	0.31 ± 0.17	-2.77	0.00	●
56402	1.00	0.77 ± 0.42	-2.97	0.00	●	1.00	0.10 ± 0.30	-16.16	0.00	●	1.00	0.50 ± 0.50	-5.39	0.00	●
58602	0.33	0.22 ± 0.23	-2.57	0.01	●	0.33	0.19 ± 0.22	-3.50	0.00	●	0.67	0.41 ± 0.21	-6.71	0.00	●
60002	0.33	0.26 ± 0.25	-1.65	0.05		0.00	0.09 ± 0.15	3.25	1.00		0.33	0.20 ± 0.24	-3.03	0.00	●
64702	0.00	0.27 ± 0.28	5.11	1.00		0.00	0.07 ± 0.17	2.11	0.98		1.00	0.15 ± 0.23	-19.98	0.00	●
75202	0.00	0.03 ± 0.08	2.11	0.10		0.00	0.03 ±0.10	1.36	0.91		0.00	0.11 ± 0.14	4.18	1.00	
80702	0.67	0.50 ± 0.20	-4.35	0.00	●	0.33	0.48 ± 0.21	3.79	1.00		0.67	0.4 ± 0.22	-6.60	0.00	●
85202	0.00	0.05 ± 0.15	1.80	0.96		0.50	0.03 ± 0.12	-20.15	0.00	●	0.50	0.07 ± 0.17	-13.73	0.00	●
93402	0.00	0.58 ± 0.32	9.87	1.00		0.00	0.88 ± 0.25	19.20	1.00		0.00	0.75 ± 0.31	13.05	1.00	
93902	0.33	0.13 ± 0.18	-5.83	0.00	●	0.00	0.00 ± 0.00	-	-		0.33	0.30 ± 0.29	-0.62	0.27	
94402	0.00	0.39 ± 0.27	7.77	1.00		0.00	0.13 ± 0.18	3.75	1.00		0.25	0.35 ± 0.24	2.44	0.99	
95202	0.50	0.19 ± 0.21	-7.87	0.00	●	0.00	0.07 ± 0.13	2.80	1.00		0.25	0.21 ± 0.18	-1.22	0.11	
96002	0.25	0.61 ± 0.25	7.80	1.00		0.25	0.49 ± 0.22	5.95	1.00		0.50	0.69 ± 0.25	4.17	1.00	
98102	0.50	0.05 ± 0.15	-16.16	0.00	●	1.00	0.03 ± 0.12	-41.74	0.00	●	1.00	0.07 ± 0.17	-29.57	0.00	●
98202	0.00	0.01 ± 0.05	1.44	0.92		0.00	0.00 ± 0.00	-	-		0.00	0.00 ± 0.00	-	-	
101702	0.50	0.28 ± 0.28	-4.18	0.00	●	0.00	0.13 ± 0.22	3.25	1.00		0.50	0.25 ± 0.25	-5.39	0.00	●
102202	0.50	0.13 ± 0.12	-15.83	0.00	●	0.00	0.01 ± 0.04	1.00	0.84		0.50	0.12 ± 0.12	-16.55	0.00	●
104602	0.50	0.23 ± 0.28	-5.11	0.00	●	0.00	0.17 ± 0.24	3.81	1.00		1.00	0.28 ± 0.31	-12.54	0.00	●
109502	0.00	0.47 ± 0.50	5.04	1.00		1.00	0.7 ± 0.46	-3.53	0.00	●	1.00	0.67 ± 0.47	-3.81	0.00	●
112802	0.33	0.42 ± 0.27	1.76	0.96		0.33	0.11 ± 0.16	-7.62	0.00	●	0.33	0.42 ± 0.26	1.86	0.96	
113902	0.67	0.78 ± 0.18	3.34	0.99		0.00	0.00 ± 0.00	-	-		1.00	0.70 ± 0.22	-7.45	0.00	●
114702	0.20	0.27 ± 0.20	1.94	0.97		0.00	0.04 ± 0.08	2.69	1.00		0.00	0.15 ± 0.18	4.43	1.00	
114902	0.25	0.01 ± 0.04	-29.00	0.00	●	0.25	0.03 ± 0.08	-13.73	0.00	●	0.50	0.02 ± 0.06	-41.74	0.00	●
123902	0.00	0.00 ± 0.00	-	-		0.00	0.00 ± 0.00	-	-		0.00	0.00 ± 0.00	-	-	
**Total**	-	-	-	-	18<br>48.65%	-	-	-	-	12<br>32.43%	-	-	-	-	23<br>62.16%
**Average ± s.d.**	0.37 ± 0.36	0.28 ± 0.25	-	-	-	0.24 ± 0.37	0.16 ± 0.19	-	-	-	0.28 ± 0.25	0.28 ± 0.26	-	-	-

Table S4: Pairwise comparison results for SS values using the multiple comparison Tukey HSD test.

Group A	Group B	Lower Limit	Difference (A-B)	Upper Limit	P-value
Control	Chronological	-0.08	0.13	0.33	0.30
Control	Cumulative	-0.33	-0.12	0.08	0.33
Chronological	Cumulative	-0.45	-0.25	-0.05	0.01

Table S5: Pairwise comparison results for FPR/h values using the multiple comparison Tukey HSD test.

Group A	Group B	Lower Limit	Difference (A-B)	Upper Limit	P-value
Control	Chronological	0.18	1.35	2.51	0.02
Control	Cumulative	-1.20	-0.03	1.14	1.00
Chronological	Cumulative	-2.55	-1.38	-0.21	0.02

5 Discussion

5.1 The influence of the number of testing seizures in the models' performance

Seizure sensitivity might be influenced by the number of tested seizures. For patients with only one seizure, the model's sensitivity is limited to 0 or 1, indicating whether the seizure is correctly predicted or not. However, for patients with multiple seizures, the model is capable of achieving different SS values. Furthermore, for patients with an elevated number of testing seizures, it might be more difficult to predict all seizures and achieve a seizure sensitivity of 1.

The following figures present the interaction between SS values and the number of testing seizures. Upon inspection, it is evident that the models achieved an SS value of 1 when the number of tested seizures was reduced. Moreover, more patients accomplished an SS value of 1 when only one seizure was tested. A general decrease in seizure sensitivity is observed as more seizures are tested.

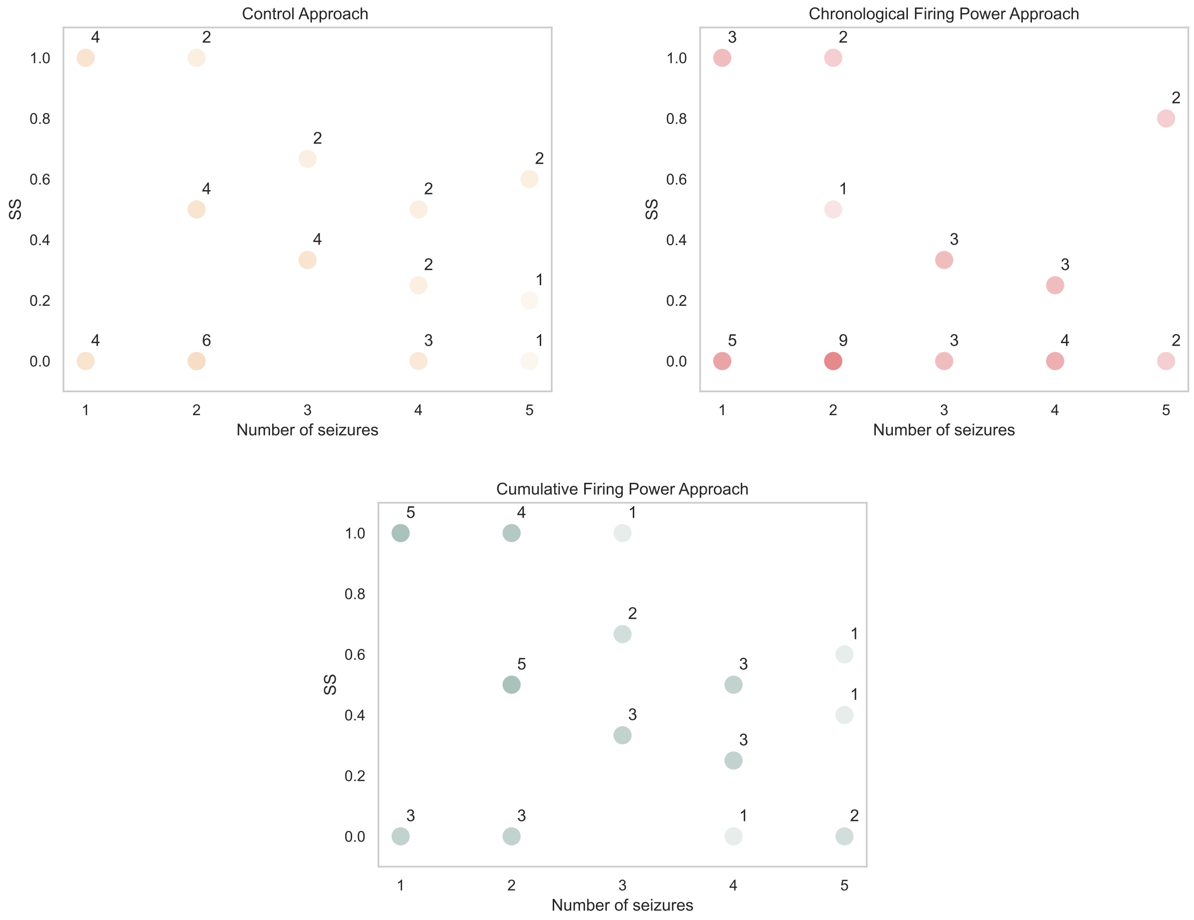

Fig. S1. Interaction between seizure sensitivity (SS) and the number of testing seizures for each approach. Near to each point is the number of models that it represents.

References

1. Klatt J, Feldwisch-Drentrup H, Ihle M, Navarro V, Neufang M, Teixeira C, et al. The EPILEPSIAE database: An extensive electroencephalography database of epilepsy patients. Epilepsia. 2012 9;53(9):1669-76.
2. Mormann F, Andrzejak RG, Elger CE, Lehnertz K. Seizure prediction: the long and winding road. Brain. 2007 2;130(2):314-33.
3. Teixeira CA, Direito B, Bandarabadi M, Le Van Quyen M, Valderrama M, Schelter B, et al. Epileptic seizure predictors based on computational intelligence techniques: A comparative study with 278 patients. Computer Methods and Programs in Biomedicine. 2014 5;114(3):324-36.
4. Aarabi A, He B. Seizure prediction in patients with focal hippocampal epilepsy. Clinical Neurophysiology. 2017;128(7):1299-307.
5. Direito B, Teixeira CA, Sales F, Castelo-Branco M, Dourado A. A Realistic Seizure Prediction Study Based on Multiclass SVM. International Journal of Neural Systems. 2017 5;27(03):1-15.
6. Mormann F, Kreuz T, Rieke C, Andrzejak RG, Kraskov A, David P, et al. On the predictability of epileptic seizures. Clinical Neurophysiology. 2005 3;116(3):569-87.
7. Rasekhi J, Mollaei MRK, Bandarabadi M, Teixeira CA, Dourado A. Preprocessing effects of 22 linear univariate features on the performance of seizure prediction methods. Journal of Neuroscience Methods. 2013 7;217(1-2):9-16.
